# Isolated fractures of the greater tuberosity: When are they treated conservatively? A baseline study

**DOI:** 10.1007/s11678-018-0459-z

**Published:** 2018-05-15

**Authors:** Benedikt Schliemann, Lukas F. Heilmann, Michael J. Raschke, Helmut Lill, J. Christoph Katthagen, Alexander Ellwein

**Affiliations:** 1 Department of Trauma, Hand and Reconstructive Surgery, University Hospital Münster, Albert-Schweitzer-Campus, building W1, 48149 Münster, Germany; 2 Traumatology and Reconstructive Surgery, Diakovere Friederikenstift GmbH, Humboldtstr 5, 30169 Hannover, Germany

**Keywords:** Greater tuberosity fracture, Shoulder dislocation, Proximal humeral fractures, Concomitant lesions, Surgery, Tuberculum-majus-Fraktur, Schulterluxation, Proximale Humerusfrakturen, Begleitverletzungen, Operation

## Abstract

**Background:**

This study analyzed the indications for conservative management of isolated greater tuberosity (GT) fractures. The rate of secondary interventions for failed conservative treatment was also assessed.

**Methods:**

A retrospective data evaluation of isolated GT fractures was performed from the clinical database of two level-I trauma centers from January 2010 to June 2017. Conservatively treated GT fractures were identified and subdivided according to etiology, morphology, and amount of initial displacement. Secondary surgical interventions were recorded and subcategorized into fracture-associated interventions and interventions for associated soft tissue lesions.

**Results:**

We identified 114 fractures. Nine cases were excluded because patients refused surgery or their comorbidities ruled it out. Only two of the remaining 105 patients had an initial displacement >3 mm. All other GT fractures (*n* = 103, 98%) were not displaced or only slightly displaced (0–3 mm). The fracture was associated with an anterior shoulder dislocation in 39 cases (37%); 17 patients (16.2%) underwent surgery after primary conservative treatment. Four of these 17 patients presented with a secondary displacement of the GT fragment. In all other cases (76.5%), an associated soft tissue lesion necessitated revision surgery. Young age, anterior shoulder dislocation, and concomitant injuries were risk factors for revision surgery after primary conservative treatment.

**Conclusion:**

Secondary interventions are required more frequently after shoulder dislocation. Surgery is most likely required for associated soft tissue lesions rather than for secondary displacements. Thus, detailed physical examination and magnetic resonance imaging should be used to screen for concomitant soft tissue injuries accompanying GT fractures to prevent revision surgeries.

## Zusammenfassung

**Hintergrund:**

Ziel der vorliegenden Arbeit war es, die Indikationen für eine konservative Behandlung bei isolierten Frakturen des Tuberculum majus (TM) zu ermitteln. Darüber hinaus wurde die Rate sekundärer chirurgischer Interventionen bei Versagen der konservativen Therapie erfasst.

**Methoden:**

Es erfolgte eine retrospektive Auswertung der Datenbanken zweier Level-I-Traumazentren, in denen isolierte TM-Frakturen von Januar 2010 bis Juni 2017 erfasst wurden. Die konservativ behandelten Frakturen wurden nach ihrer Ätiologie sowie der Morphologie und Dislokation des Fragments ausgewertet und unterschieden. Sekundäre chirurgische Interventionen wurden erfasst und ebenfalls weiter unterschieden in frakturassoziierte Maßnahmen und in Interventionen, die aufgrund von mit Weichteilverletzungen erforderlich wurden.

**Ergebnisse:**

Es wurden 114 TM-Frakturen identifiziert. Neun Fälle wurden aus der Studie ausgeschlossen, weil die Patienten die empfohlene operative Therapie ablehnten oder diese aufgrund von Komorbiditäten nicht möglich war. Bei nur 2 der verbliebenen 105 Patienten war die Fraktur >3 mm disloziert, alle anderen TM-Frakturen (*n* = 103, 98 %) waren initial nicht oder nur leicht disloziert (0–3 mm). In 39 Fällen (37 %) war die Fraktur mit einer anterioren Schulterluxation assoziiert. Bei 17 Patienten (16,2 %) erfolgte nach initialer konservativer Therapie eine sekundäre chirurgische Intervention. Bei 4 dieser 17 Patienten war die Revision wegen einer sekundären Dislokation des TM-Fragments notwendig. In allen anderen Fällen (76,5 %) machte eine assoziierte Weichteilverletzung die sekundäre Operation erforderlich. Junges Patientenalter, eine anteriore Schulterluxation und begleitende Weichteilverletzungen stellten Risikofaktoren dar, welche die Wahrscheinlichkeit für eine sekundäre chirurgische Intervention nach primär konservativer Therapie erhöhten.

**Schlussfolgerung:**

Sekundäre chirurgische Interventionen sind häufiger nach anteriorer Schulterluxation und mit größerer Wahrscheinlichkeit bei begleitenden Weichteilverletzungen als bei sekundären Dislokationen notwendig. Daher sollte durch eine detaillierte körperliche Untersuchung und ggf. durch eine MRT der Fokus auf die Suche nach begleitenden Weichteilverletzungen bei TM-Frakturen gelegt werden, um Revisionsoperationen vorzubeugen.

Fractures of the greater tuberosity (GT) often occur with more complex proximal humerus fractures and are less frequently observed as an isolated pathology. Only 14–20% of proximal humerus fractures are isolated lesions of the GT [4, 15, 23]. Up to 30% of these fractures are associated with anterior glenohumeral dislocations [25].

According to Neer, a displacement of the fragment of >10 mm and 45° (later modified to >5 mm and 30°) is believed to be an indication for operative treatment [20]. However, whether all other fractures can be managed successfully by nonoperative treatment is unclear. Currently, there is a lack of evidence in the literature to support either conservative or operative treatment strategies in GT fractures. Whether the fracture type and the etiology of the fracture impact the decision-making and the final outcome also remains unclear.

There are only a few reports on conservative treatment of isolated GT fractures. Platzer et al. compared the functional results of 52 patients who underwent open reduction and internal fixation (ORIF) of a GT fracture with nine patients who were treated conservatively for similar fractures. All patients had a displaced fracture (>5 mm) [23]. After a mean follow-up of 5.5 years, functional results were significantly better after operative treatment than they were after conservative treatment. The same authors reported on 135 conservatively treated patients with an isolated GT fracture and found worse results in fractures with >3 mm displacement [22]. Similarly, other authors reported good to excellent results in their patients who had conservative treatment for minimally displaced fractures [12, 17, 24].

The present baseline study aimed to analyze under what circumstances isolated fractures of the GT are managed conservatively. In addition to the indication for conservative treatment, the rate of secondary interventions for failed conservative treatment is evaluated.

## Patients and methods

A retrospective data evaluation of isolated GT fractures was performed from the clinical database of two german level-I trauma centers from January 2010 to June 2017. A total of 114 patients with a GT fracture were initially treated conservatively. The mean age of the patients at the time of the injury was 55 years (range, 18–94 years). The mechanism of the injury was a direct fall on the affected shoulder in 61 cases, a traffic accident in nine cases, and an anterior shoulder dislocation in 44 cases. All patients had conventional radiographs of the shoulder in the anteroposterior, axillary, and Y‑view. After an isolated fracture of the GT was identified, the degree of displacement was assessed and classified as: (1) no displacement, (2) mild displacement (1–3 mm), or (3) severe displacement (>3 mm). Impression fractures (usually related to glenohumeral dislocations) comprised a fourth group. The fractures were further subdivided into simple fractures with only a single fragment and comminuted fractures with two or more fragments.

The indication for conservative treatment was analyzed according to the patients’ records. A follow-up period of at least 6 months was mandatory in order to evaluate secondary surgical interventions. Interventions were further subdivided into fracture-related procedures and procedures required for associated lesions, such as capsulolabral tears in patients who sustained a shoulder dislocation or posttraumatic stiffness.

## Results

**Fig. 1:**
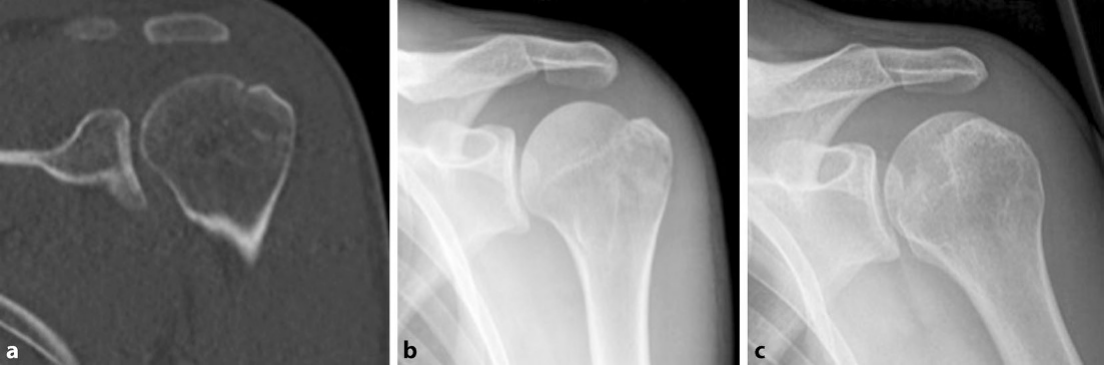
Conservatively treated fracture of the greater tuberosity: initial radiograph (**a**), follow-up after 3 weeks to exclude secondary displacement (**b**), and anatomic consolidation 3 months after the injury (**c**)

A total of 114 patients were identified. In six patients with severe displacement, operative treatment was recommended, but severe comorbidities (i.e. cardiovascular conditions) prevented surgical interventions. In addition, two patients refused to undergo surgery despite severe concomitant soft tissue lesions, and one patient did not return to the hospital after an magnetic resonance imaging (MRI) was indicated. These nine patients were excluded from the study. Of the remaining 105 fractures, 72 (68.6%) were not displaced (Fig. 1), a mild displacement of 1–3 mm was found in 27 cases (25.7%), severe displacement (>3 mm) was found in two cases (1.9%), and the remaining four fractures (3.8%) were classified as impression fractures (depression type fracture). The decision to treat the two cases with more severe displacement conservatively was made based on an initial displacement of 4 mm. 51% of the fractures were simple and 49% had multiple fragments. Comminuted fractures were more likely to occur with shoulder dislocations (57%). In the acute situation, all fractures were treated conservatively with the affected arm immobilized in an abduction brace to release tension from the rotator cuff on the fracture site. Passive motion was allowed with range of motion (ROM) limited to 90° of flexion and abduction, free external rotation, and no internal rotation for the first 3 weeks.

Patients were reexamined clinically and radiographically to identify secondary displacements. If the patient had further complaints, MRI was performed to analyze the integrity of the rotator cuff and the capsuloligamentous complex.

**Fig. 2:**
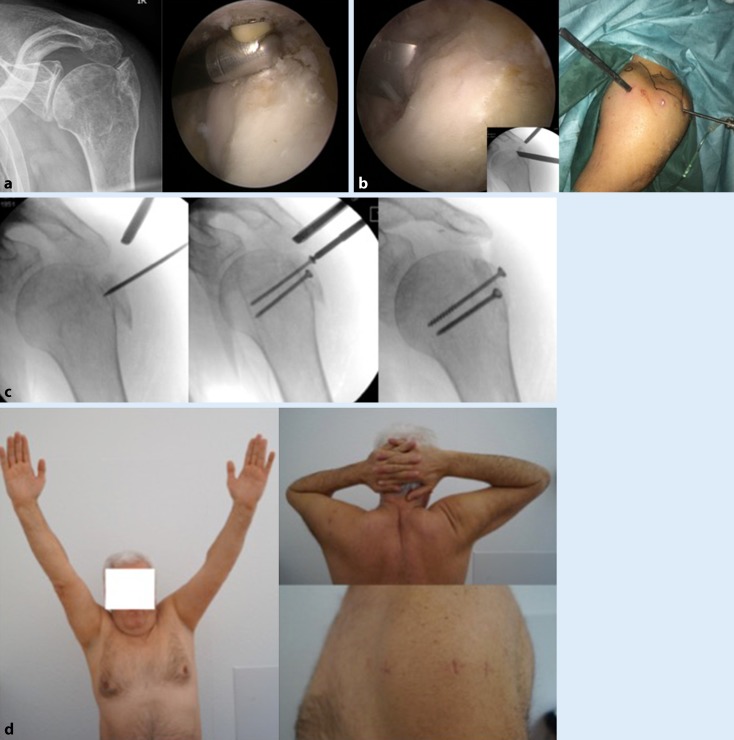
**a** Posterosuperior malunion of a solid greater tuberosity fracture. Preoperative anteroposterior radiograph (*left*) and intraoperative finding (*right*). **b** Osteotomy of the fragment (*left*) with corresponding fluoroscopy (*inset*) and external view (*right*). **c** Under arthroscopic and fluoroscopic guidance, the fragment is reduced and fixed with two 3.5-mm screws. **d** Functional outcome four months postoperatively

Of the remaining 105 patients, 17 underwent secondary surgical interventions (16.2%). In only four cases, including the two patients with initial displacement of the GT fragment >3 mm, a surgical revision was required owing to a secondary displacement of the fracture with subsequent impingement and limited ROM (Fig. 2). In all other cases (*n* = 13), an associated soft tissue lesion led to a secondary surgical intervention. The different procedures are listed in Table 1. Given that three patients refused to undergo surgery despite an indication because of secondary displacement and subsequent limited ROM, the revision rate would have increased to 19%.

**Table 1:** Overview of secondary surgical interventions

Patient age	Gender	Displacement diameter	Trauma mechanism	Reason for secondary surgery	Surgical intervention	Time point of revision surgery^a^
77	Female	4 mm (cranial)	Traumatic shoulder dislocation	Secondary displacement	ORIF PHILOS plate	1
80	Female	4 mm (cranial)	Fall	Secondary displacement	ORIF PHILOS plate	2.5
28	Male	No displacement	Traffic accident	Secondary displacement	Twinfix anchor	4.5
46	Female	No displacement	Traumatic shoulder dislocation	Secondary displacement + LHBT tendinitis	A. subacromial decompression + LHBT tenodesis	21
64	Male	No displacement	Traumatic shoulder dislocation	LHBT tendinitis + SSP rupture	LHBT tenodesis + SSR	13.5
44	Female	3 mm (lateral)	Fall	Malposition of healed fracture + LHBT tendinitis + SSP rupture	LHBT tenodesis, tuberculoplasty, SST repair	84
21	Male	No displacement	Traumatic shoulder dislocation	Bankart lesion	A. labral repair and capsular shift	54
25	Male	1 mm (cranial)	Traumatic shoulder dislocation	Bankart lesion + PTSS	A. arthrolysis, labral repair and capsular shift	20
43	Male	No displacement	Traumatic shoulder dislocation	PTSS	A. arthrolysis	16
55	Male	No displacement	Traumatic shoulder dislocation	PTSS	A. arthrolysis + LHBT tenodesis + acromioplasty	24
56	Female	No displacement	Fall	PTSS + LHBT tendinitis	A. arthrolysis + LHBT tenodesis	25
57	Female	No displacement	Traffic accident	PTSS + Impingement	A. arthrolysis + acromioplasty	19
45	Male	No displacement	Fall	SST, SLAP, LHBT rupture	LHBT tenodesis + SSR	25
35	Male	No displacement	Fall	SST	SSR + LHBT tenodesis	28
43	Female	No displacement	Fall	SST	SSR	24
62	Female	No displacement	Fall	Impingement	Subacromial decompression	8
29	Male	3 mm (cranial)	Traumatic shoulder dislocation	Axillary nerve injury	Neurolysis + decompression brachial plexus	30

^a^Weeks after trauma

*PTSS* posttraumatic shoulder stiffness, *A. a*rthroscopic, *ORIF* open reduction and internal fixation, *LHBT* long head biceps tendon, *SST* supraspinatus tear, *SSR* supraspinatus repair, *SLAP* superior labral tear from anterior to posterior

Patients for whom a shoulder dislocation was the cause of the GT fracture were more likely to undergo secondary surgical intervention (20.5% vs. 13.6%). However, this difference was not statistically significant (*p* = 0.415).

The fracture morphology (single fragment vs. comminuted fractures) had no significant impact on the risk for secondary interventions.

By contrast, the amount of the initial displacement of the fragment is an indicator of secondary displacement and surgical revisions; all patients with primary displacement of the GT fragment who were treated conservatively and needed revision surgery because of a secondary displacement of the GT fragment had a significant primary displacement of ≥3 mm.

Moreover, younger patients tend to require secondary interventions more frequently than older patients. The mean age of the patients who underwent surgical intervention was 46 years (25–62 years), whereas the mean age of the cohort without secondary intervention was 56 years (18–94 years). However, this difference was not statistically significant (*p* = 0.148).

## Discussion

The present database analysis revealed three major findings: First, the majority of conservatively treated isolated GT fractures (68.6%) are nondisplaced, generally justifying conservative treatment. Second, the rate of secondary interventions was 16.2%. Finally, most of the secondary surgical interventions were not related to the secondary displacement of the GT fracture but to concomitant lesions of the rotator cuff and the capsuloligamentous tissue.

According to the criteria originally defined by Neer, a displacement of >10 mm and 45° is an indication for surgery, and all other fractures can be successfully managed with a conservative approach [20]. Later, these criteria were modified to 5 mm and 30° of displacement. However, it is known that as little as 2–5 mm of displacement can cause impingement and requires increased forces for abduction [7–9]. In particular, fragment displacement in the posterosuperior direction is associated with impaired function and worse results [2, 27]. Therefore, operative treatment is recommended more aggressively, and numerous articles exist about different techniques and results [1, 3, 5, 6, 13, 17, 23, 26].

By contrast, there are only a few reports on conservative treatment of isolated GT fractures. Platzer et al. reported on the functional results of 135 patients treated conservatively for isolated GT fractures with less than 6 mm displacement [22]. They found good to excellent results in 97% of the cases. This is in accordance with results from other studies of conservative treatment [12, 17, 20, 23, 24]. If the displacement is less than 5 mm, satisfying results can be expected. In addition, the present study shows that patients with a nondisplaced or only slightly displaced fracture (0–3 mm) that did not arise from a shoulder dislocation have a low risk for secondary surgical interventions. Furthermore, fractures with an initial displacement of 3 mm or less are unlikely to have further displacement over time (only 2% in the present study). Patients who required secondary surgery after initial displacement had a primary fracture displacement of >3 mm.

Unfortunately, defining the degree of displacement has also been a matter of debate. When displacement is measured with only plain radiographs, errors of up to 13 mm have been described [21]. A computed tomography (CT) scan may help to minimize these errors. On the other hand, Janssen et al. observed that the imaging modality did not influence the reliability of the fracture assessment or the recommendation for surgical treatment [11]. Mutch et al. suggested using a greater tuberosity ratio (GT ratio) that can be applied to plain radiographs [19]. They found a very strong correlation with computed tomography (CT) scans for superior GT displacement. Furthermore, the GT ratio helped to accurately identify fractures as suitable for conservative or operative treatment or as benefitting from further imaging.

In addition, the amount of initial displacement is relevant to the decision on the treatment modality. However, there is an immediate need to reevaluate patients treated conservatively, since 50–60% of fractures show further displacement over time [22]. Younger patients are at an especially heightened risk. Hebert-Davies et al. found a 5.6-fold higher risk for secondary displacement in patients younger than 70 years compared with patients over 70 years of age [10]. Similarly, in our study, patients with secondary displacement and surgical intervention were younger than the mean age of the cohort (46 vs. 55 years).

Therefore, both aspects, the degree of displacement and how to adequately assess it, must be further investigated.

Another major finding of the present analysis is that concomitant soft tissue lesions lead to a secondary intervention after initial conservative treatment in over three quarters of the cases. In the present analysis, surgical intervention owing to secondary displacement was only performed in four cases (3.8%). Other common interventions include capsuloligamentous and rotator cuff repairs (Table 1). These findings highlight the need for further imaging, particularly MRI scans, to detect any concomitant lesions. Especially in patients with anterior shoulder dislocation and multi-fragmentary GT fracture, concomitant lesions are frequently found and require further operative treatment. Maman et al. reported on 24 arthroscopically treated patients with a GT fracture. Concomitant soft tissue lesions were found in 22 patients (94%) [16]. These findings are supported by Katthagen et al., who found concomitant lesions (i. e., pulley/SLAP and Bankart lesions) in 69% of patients who were treated arthroscopically for a GT fracture [14]. Again, these lesions were found more frequently after shoulder dislocations.

Muhm et al. found concomitant lesions in GT fractures with and without a dislocation [18]. However, in patients with a dislocation, concomitant lesions were more likely to be treated operatively. Interestingly, in the Muhm study, GT fractures with three or more fragments were always associated with anterior shoulder dislocation. In the present analysis, complex fracture patterns were found even in patients without previous shoulder dislocation, although patients were more likely to have a multi-fragmentary fracture when they sustained a shoulder dislocation. The risk for secondary surgical interventions increased with dislocations but not with multi-fragmentary fracture patterns.

### Limitations

Some inherent limitations apply to the present analysis. Only patient records were analyzed, and the final functional and radiographic outcomes remain unclear in most cases. Therefore, we cannot provide proof of whether or not conservative treatment leads to good results in patients without secondary interventions. Furthermore, the decision to apply conservative treatment was not based on a distinct algorithm. There is a clear trend, however, toward conservative treatment in patients with only minimally displaced fractures. In most cases, patients with severely displaced fractures were treated conservatively when there were contraindications for surgery or when patients refused to undergo surgical treatment.

Finally, imaging modalities were not consistent in all the cases since not every patient received a CT and/or MRI scan before the decision to apply conservative treatment was made.

## Practical conclusion

- With the exception of two cases, all patients included in this study had no displacement or only slight displacement of the GT fragment (0–3 mm) and a low risk for secondary surgical interventions.
- Secondary interventions are required more frequently after shoulder dislocation. In addition, revision surgery is most likely required because of concomitant soft tissue lesions rather than prompted by secondary displacements.
- Detailed physical examination and MRI scans should be utilized in order to screen for concomitant soft tissue injuries accompanying GT fractures so as to prevent revision surgeries.
- The study highlights the need for further prospective studies in order to define clear indications for conservative treatment based on fracture patterns, imaging modalities, concomitant lesions, and the patients’ individual requests.
